# Supplementary material for: High resolution ancient sedimentary DNA shows that alpine plant diversity is associated with human land use and climate change
Source: Nat Commun. 2022 Nov 4;13:6559. doi: 10.1038/s41467-022-34010-4 (PMC9636257; doi:10.1038/s41467-022-34010-4)
Supplement: Supplementary file 1 — Supplementary Information [file 41467_2022_34010_MOESM1_ESM.pdf]

Nature communications Supplementary Information

Article title: *High resolution ancient sedimentary DNA shows that alpine plant diversity is associated with human land use and climate change*

Authors: *Sandra Garcés-Pastor*, Eric Coissac, Sébastien Lavergne, Christoph Schwörer, Jean-Paul Theurillat, Peter D. Heintzman, Owen S. Wangensteen, Willy Tinner, Fabian Rey, Martina Heer, Astrid Rutzer, Kevin Walsh, Youri Lammers, Antony G. Brown, Tomasz Goslar, Dilli P. Rijal, Dirk N. Karger, Loïc Pellissier, PhyloAlps Consortium, Oliver Heiri#, Inger Greve Alsos#*

Supplementary Tables

Table 1: Radiocarbon dates of Sulseewlii according to depths of the compound core …….………………..… 1

Table 2: *Seda*DNA sequences used to infer vegetation belts (87 out of 366) and the equivalent pollen taxa found within the Sulsseewli sediments ……………………………………………...………..………………. 3

Table 3: Plant *seda*DNA sequences identified with each database ……………..…………………….………….. 4

Table 4: Suitable indicator taxa identified by each database in order of priority ………………………………. 4

Table 5: Taxa identified with PhyloAlps that are present in other databases …………………….………..…..… 4

Table 6: *Seda*DNA used to infer pastoral activities and equivalent pollen taxa in the Sulsseewli sediments … 4

Table 7: Summary of the plant *seda*DNA results ………………………………………………………………….……… 5

Table 8: Summary of the mammal *seda*DNA results . ………………………………………………..……………... 5

Table 9: Spearman's rank correlations between samples passing relaxed/strict QC and richness/time for six measures of *seda*DNA data quality . ……………………………………………………………………..………… 6

Table 10: Summary statistics for generalised additive models (GAMs) fitted after outlier removal ………….... 7

Table 11: Complete outcomes of the fitted model tested in the Piecewise structural equation model (SEM) ... 8

Supplementary Figures

Figure 1: Core correlation of SUL A-B-C ………………………………………….…….……………………..….. 9

Figure 2. Age-depth model of the composite core ………………………………..…………………..……….….. 10

Figure 3: Chironomid record from Sulsseewli ……………………………………………..………….…..…..…. 11

Figure 4: Proportion of weighted PCR replicates of plant *seda*DNA ………………..…………………..……. 12

Figure 5: Pollen diagram of Sulsseewli ……………………………………………..………………..…….….…. 16

Figure 6: Summary of the indicators for human activities in the Sulsseewli sediments ..…………..……......…. 17

Figure 7: Percentages of functional groups inferred by *seda*DNA and pollen …………..………....……..… 18

Figure 8: Richness of *seda*DNA within different plant functional groups ……………..………..……………….. 19

Figure 9: All retained animal *seda*DNA detections ………………………..……………………...………...….. 20

Figure 10: Correlations between total taxonomic richness and time against six measures of plant *seda*DNA data quality ………………………………………………………………..…………………………….… 21

Figure 11: Proportion of all reads before and after the plant metabarcoding data quality control …………..… 22

Figure 12: Redundancy analysis (RDA) of the plant species composition and elevational vegetation belts from *seda*DNA across the record constrained by 12 explanatory variables ………………….. 23

Figure 13: Piecewise redundancy analysis …………………………………………..……………..…..………….. 24

Figure 14: Distribution of residuals before and after the removal of the outlier from generalised additive models (GAMs) ……………………………………………………………………………………………. 25

Figure 15: Full model used for structural equation model analysis …………………..……………………….. 26

Supplementary Table 1. Radiocarbon dates of Sulseewlii according to depths of the compound core. Macroremains were dated in Poznan (Poz) and Bern (BE) laboratories.

Lab. code	Depth [cm]	Material	Core segment	14C age [BP]	Median calibrated age [cal BP]
**BE-12772.1.1**	102−106	*Betula* fruit, *Picea abies* twig	A (0-1 m)	560 ± 20	556
**BE-12773.1.1**	148−152	*Dryas octopetala* leaf fragments	A (1-2 m)	1000 ± 30	913
**Poz-111382**	197-198	Needle	C (2,4-3 m)	1380 ± 50	1297
**BE-9756.1.1**	239−241	Twig	B (2-3 m)	1940 ± 20	1863
**Poz-123903**	296-297	Needle	C (3-4 m)	2600 ± 80	2699
**Poz-123857**	305-306	Branch	C (3-4 m)	2825 ± 30	2926
**BE-10808.1.1**	307−309	*Picea abies* needles	B (3-4 m)	2830 ± 20	2929
**Poz-111381**	318-319	Branch	C (3-4 m)	2945 ± 35	3103
**Poz-123856**	323-324	Needle	C (3-4 m)	3045 ± 35	3255
**Poz-111380**	331-332	Branch	C (3-4 m)	3085 ± 35	3292
**BE-9755.1.1**	365.5−367.5	*Picea abies* needle, *Picea abies* seeds, *Pinus cembra* needle	A (4-5 m)	3625 ± 20	3934
**BE-10809.1.1**	412.5−414.5	*Picea abies* needle, *Picea abies* seeds, coniferous bud scale	A (4-5 m)	4135 ± 20	4681
**Poz-111508**	427-428	Wood	C (4-5 m)	4085 ± 30	4580
**BE-12775.1.1**	445−447	*Pinus cembra* needles, *Pinus cembra* seed	A (5-6 m)	4690 ± 25	5387
**Poz-111507**	452-453	Wood	C (4-5 m)	4885 ± 35	5623
**BE-9754.1.1**	477−479	Bark	A (5-6 m)	5590 ± 20	6354
**BE-12776.1.1**	493−495	Bark	A (5-6 m)	6470 ± 25	7368
**BE-12777.1.1**	509−511	*Betula* fruit scale, *Pinus cembra* needles	A (5-6 m)	7325 ± 25	8101
**BE-10810.1.1**	521.5−523.5	Twig	A (5-6 m)	8220 ± 25	9188

**BE-12778.1.1**	533−535	*Betula* fruit scale, cone scale	A (5-6 m)	8625 ± 40	9588
**BE-9753.1.1**	555−557	Bud scale, short shoot, twigs	A (6-7 m)	9315 ± 25	10527
**BE-12779.1.1**	568−572	*Dryas octopetala* leaf fragments, fruit stand	A (6-7 m)	9650 ± 60	11003
**Poz-138479**	608.5-609.5	Terrestrial plant remains	C (6-7 m)	10590 ± 250	12432

Supplementary Table 2. *Seda*DNA sequences used to infer vegetation belts (87 out of 366) and the equivalent pollen taxa found within the Sulsseewli sediments. Taxa identified as colline, warm colline and very warm colline based on Flora Indicativa were pooled in the category colline. See Methods section for details on this approach.

Vegetation belt	Number of taxa	DNA Taxa	Equivalent pollen taxa in the Sulsseewli record
**Alpine**	9	*Achillea atrata, Bryaceae, Carex parviflora, Crepis rhaetica, Dicranidae, Leucanthemopsis alpina, Ranunculus glacialis, Sibbaldia procumbens, Silene acaulis*	Chicorioideae, Rosaceae, Silene
**Subalpine-alpine**	14	*Androsace chamaejasme, Arctostaphylos alpina, Astragalus alpinus, Bartsia alpina, Carex frigida, Dryas octopetala, Gentiana clusii, Hedysarum hedysaroides, Hieraciinae, Pedicularis oederi, Pedicularis verticillata, Saussurea*	Androsace, *Bartsia*-type, Cyperaceae, *Dryas octopetala*, Fabaceae indet
**Subalpine**	12	*Achillea macrophylla, Allium victorialis, Arctostaphylos uva-ursi, Athamanta cretensis, Athyrium distentifolium, Botrychium, Chaerophyllum, Crepis bocconi, Delphinium elatum, Pinguicula alpina, Rhododendron, Viola biflora*	*Achillea*-type, *Allium*-type, *Athyrium filix-femina*, *Botrychium*, *Chaerophyllum*-type, Cichorioideae, *Rhododendron*
**Montane-subalpine**	22	*Asplenium, Calluna vulgaris, Carum carvi, Erica carnea, Erinus alpinus, Geranium sylvaticum, Globularia cordifolia, Lonicera alpigena, Lonicera nigra, Melampyrum sylvaticum, Meum athamanticum, Nardus stricta, Picea abies, Polystichum lonchitis, Ranunculus, Rumex scutatus, Saxifraga rotundifolia, Stellaria nemorum, Tozzia alpina, Trollius europaeus, Vicia sylvatica*	*Calluna vulgaris, Lonicera alpigena, Melampyrum, Picea abies, Ranunculus acris*-type, *Rumex acetosa*-type, *Saxifraga aizoides*-type, *Saxifraga granulata, Trollius*
**Montane**	8	*Abies alba, Chaerophyllum aureum, Chamaenerion angustifolium, Chrysosplenium alternifolium, Crepis paludosa, Ophioglossum vulgatum, Pteridium aquilinum, Saxifraga rosacea*	*Abies alba, Chaerophyllum*-type, Cichorioideae, *Pteridium aquilinum, Saxifraga aizoides*-type
**"Colline-montane"**	10	*Asplenium trichomanes, Asplenium trichomanes, Dactylorhiza incarnata, Lathyrus pratensis, Myosotis arvensis, Plantago media, Potamogeton, Sanicula, Veronica serpyllifolia*	*Cirsium, Lathyrus*-type, *Plantago media, Veronica*-type
**Colline**	5	*Acer, Cuscuta europaea, Hypochaeris maculata, Juglans regia, Tilia*	*Acer, Juglans regia, Tilia*
**Warm Colline**	5	*Castanea sativa, Fabeae, Jurinea, Lithospermeae, Orobanche*	*Castanea sativa*, Fabaceae indet
**Very warm Colline**	5	*Myrtus communis, Parentucellia latifolia, Quercus, Ranunculus parviflorus, Veronica cymbalaria*	*Quercus robur-pubescens*-type, *Ranunculus acris*-type, *Veronica*-type

Supplementary Table 3. Plant *sed*aDNA sequences identified with each database

Database	Proportion of sequences assigned to vascular plants	Proportion of vascular plants with a 100% match	Taxonomic assignment to genus or below	Identifications used in the final taxonomic assignment
**PhyloAlps**	368/375 = 98.1 %	292/368 = 79.3 %	248/292 = 84.9 %	326/375 = 86.9 %
**EMBL**	352/375 = 93.9 %	327/352 = 92.9 %	189/327 = 57.8 %	45/375 = 12 %
**PhyloNorway**	364/375 = 97 %	199/364 = 54.7 %	166/199 = 83.4 %	4/375 = 1 %
**ArctBorBryo**	353/375 = 94.1 %	181/353 = 51.3 %	154/181 = 85 %	-

Supplementary Table 4. Suitable indicator taxa identified by each database in order of priority

Database	Identified taxa
PhyloAlps	84
PhyloNorway	2
ArctBorBryo	1
EMBL	1

Supplementary Table 5. Taxa identified with PhyloAlps that are present in other databases

Only present in PhyloAlps	31
Present in 1 more database	30
Present in 2 more databases	14
Present in all databases	9

Supplementary Table 6. *Sed*aDNA used to infer pastoral activities and equivalent pollen taxa in the Sulsseewli sediments

**DNA Taxa**	**Equivalent pollen taxa in the Sulsseewli record**
Calluna vulgaris	
Carum carvi	
Cirsium arvense	*Calluna vulgaris*
Plantago major	
Plantago media	
Pteridium aquilinum	*Plantago major*
Rhinanthus alectorolophus subsp. freynii	*Plantago media*
Urtica dioica	*Pteridium aquilinum*

Supplementary Table 7. Summary of the plant *seda*DNA results.

Raw paired-end plant DNA sequences	20,517,660 sequences
After filtering and identification	7,395,214 sequences
Total samples	80
Low quality samples - relaxed	6
Low quality samples - strict	24
Total plant sequences	366
Contaminants	14 (see Supplementary Data 8)

Life-form categories and ranks assigned with plant *seda*DNA

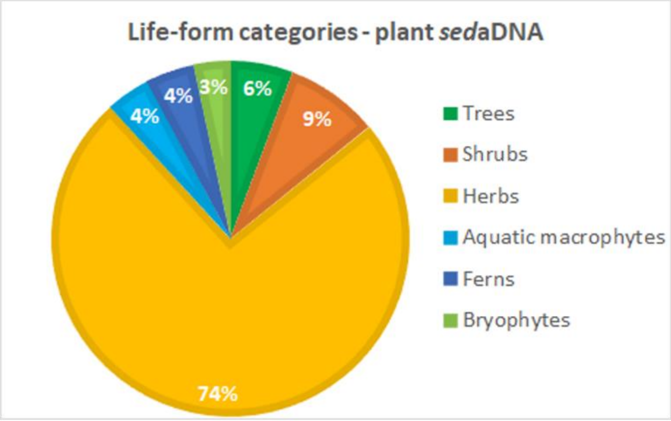

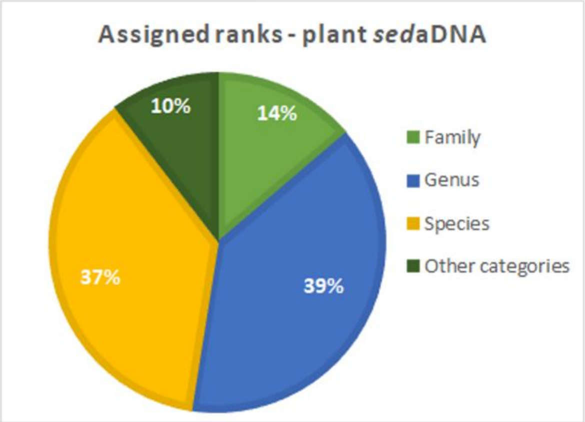

Life-form categories assigned with pollen

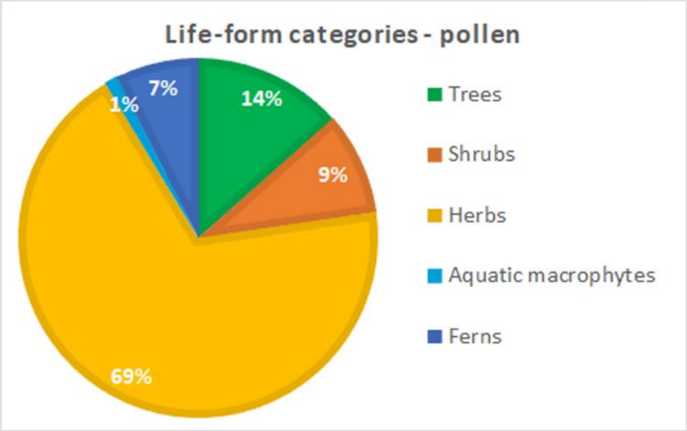

Supplementary Table 8. Summary of the mammal *seda*DNA results.

Raw paired-end mammal DNA sequences	17.5 million sequences
After filtering and identification	1,794,975 sequences
Total samples	80
Contaminants	human

Supplementary Table 9. Correlations between samples passing relaxed/strict QC and richness/time for six measures of *sed*aDNA data quality, based on a one-sided Spearman's rank correlation tests without adjustment for multiple comparisons. Tests were calculated using the cor.test function in R. Exact p-values are provided when p>2.2E-16.

	**Relaxed QC filtering**						**Strict QC filtering**					
	Richness			Time			Richness			Time		
Measure	rho	S	p	rho	S	p	rho	S	p	rho	S	p
MTQ score	0.6766602	21834	3.65E-11	-0.3505613	91197	0.002198	0.3319399	19547	0.012440	-0.03347899	30240	0.806500
MAQ score	0.7907073	14132	<2.2e-16	-0.426756	96342	0.000150	0.5988539	11738	0.000001	-0.1708108	34258	0.208100
Count of raw reads	-0.0671135	72057	0.569900	0.1288856	58822	0.273800	- 0.08236461	31670	0.546200	0.1483937	24918	0.275100
Mean barcode length	0.1310508	58676	0.265700	-0.1003636	74302	0.394900	0.1938936	23587	0.152200	0.01086808	28942	0.936600
Mean wtRep	0.8703425	8755.1	<2.2e-16	-0.6315735	110172	1.61E-09	0.7849768	6291.6	8.04E-13	-0.5032126	43984	0.000077
Prop. reads identified as terrestrial plants	0.4069617	40045	0.000320	-0.2718549	85882	0.019130	0.1518053	24818	0.264000	-0.06903623	31280	0.613200

Seven samples were removed due to low DNA quality, which had metabarcoding technical quality scores of <0.45 and/or metabarcoding analytical quality scores of <0.175, and were based on comparison to the controls (listed in Supplementary Dataset S3. These samples were L715, L724, L742, L751, L760 and L769.

Supplementary Table 10. Summary statistics for generalised additive models (GAMs) fitted after outlier removal. Taxonomic richness was treated as the response variable. Deviance explained and adjusted R square values for the full and reduced models were 80.1 and 76.7%, and 0.67 and 0.66 respectively. Data analyses performed on the strict dataset (n=56 samples, see methods).

Models	**Explanatory variables**	**Effective degrees of freedom (edf)**	**Chi square value**	**p-value**
Full	Temperature	2.1	2.6	0.4796
	Precipitation	6.5	57.2	<0.0001
	Coprophilous fungi	2.1	5.6	0.0722
	Bos taurus	1	3.3	0.0704
	Capra hircus	4.7	17.7	0.006
	Ovis aries	4.4	50.4	<0.0001
Reduced	Precipitation	6.5	59.6	<0.0001
	Capra hircus	5.6	22.6	0.002
	Ovis aries	4.1	58.4	<0.0001

Supplementary Table 11. Complete outcomes of the fitted model tested in the Piecewise structural equation model (SEM) showing the standardised coefficients of the precipitation, the wild grazing mammals and domesticates on richness. Significant paths (P < 0.05) are denoted in bold. Significance codes: "***" 0.001, "**" 0.01, "*" 0.05.

Response	Predictor	Estimate	Std. Error	DF	Crit. Value	P. Value	Std. Estimate	Significance
**Richness**	**Precipitation**	9 935	3 718	56	26722	102	**3 318**	*
**Richness**	**Cow**	3 375	1 078	56	31317	29	**3 363**	**
**Richness**	Ibex	3 179	2 379	56	13362	1 876	1 516	
**Richness**	Sheep	923	979	56	9 421	3 508	1 151	
**Richness**	Chamois	4 782	0,24	56	19927	519	2 257	
**Richness**	**Charcoal**	9	3	56	31894	25	**3 747**	**
**Cow**	Precipitation	1 157	4 057	56	2 852	7 766	388	
**Ibex**	**Precipitation**	-4 381	1 849	56	-23690	214	**-3 068**	*
**Sheep**	**Precipitation**	9 821	4 594	56	21378	372	**263**	*
**Sheep**	**Charcoal**	11	4	56	29000	54	**3 568**	**
**Charcoal**	Precipitation	2722377	1599539	56	17020	945	2 256	
**Charcoal**	**Precipitation**	-4 348	1 829	56	-23768	21	**-3 077**	2 256

Fisher's C = 6.259 with P-value = 0.995 and on 18 degrees of freedom

Response	**method**	**R squared**
Richness	none	0.45
Cow	none	0
Ibex	none	0.09
Sheep	none	0.24
Charcoal	none	0.05
Chamois	none	0.09

AIC	BIC
54.259	102.867

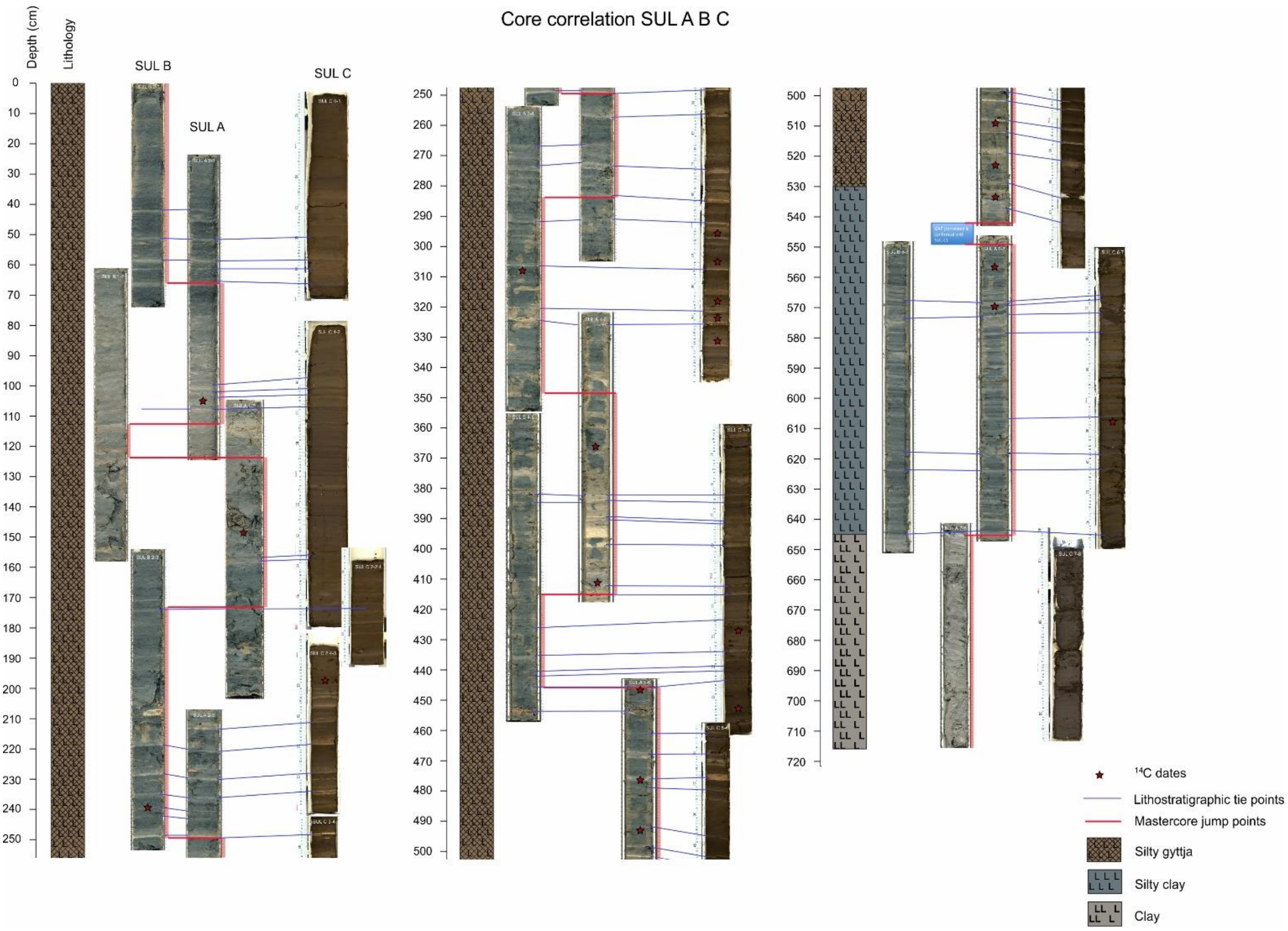

Supplementary Figure 1. Core correlation of SUL A-B-C. The composite core has three sedimentary units: unit A is composed of clay (716-645 cm), unit B of organic silt with clay (645-530 cm) and unit C of brown gyttja with silt laminations (530-0 cm). Red lines show the correlation points used to tie the master core and blue lines link the same lithostratigraphical features. Note: Colour differences between SULB and C versus SULC are because these have been photographed by different photographic equipment (SULB and C in Institute of Plant Sciences (University of Bern) and SUL C in UiT (Tromsø)) and the opened cores were at different states of oxidation.

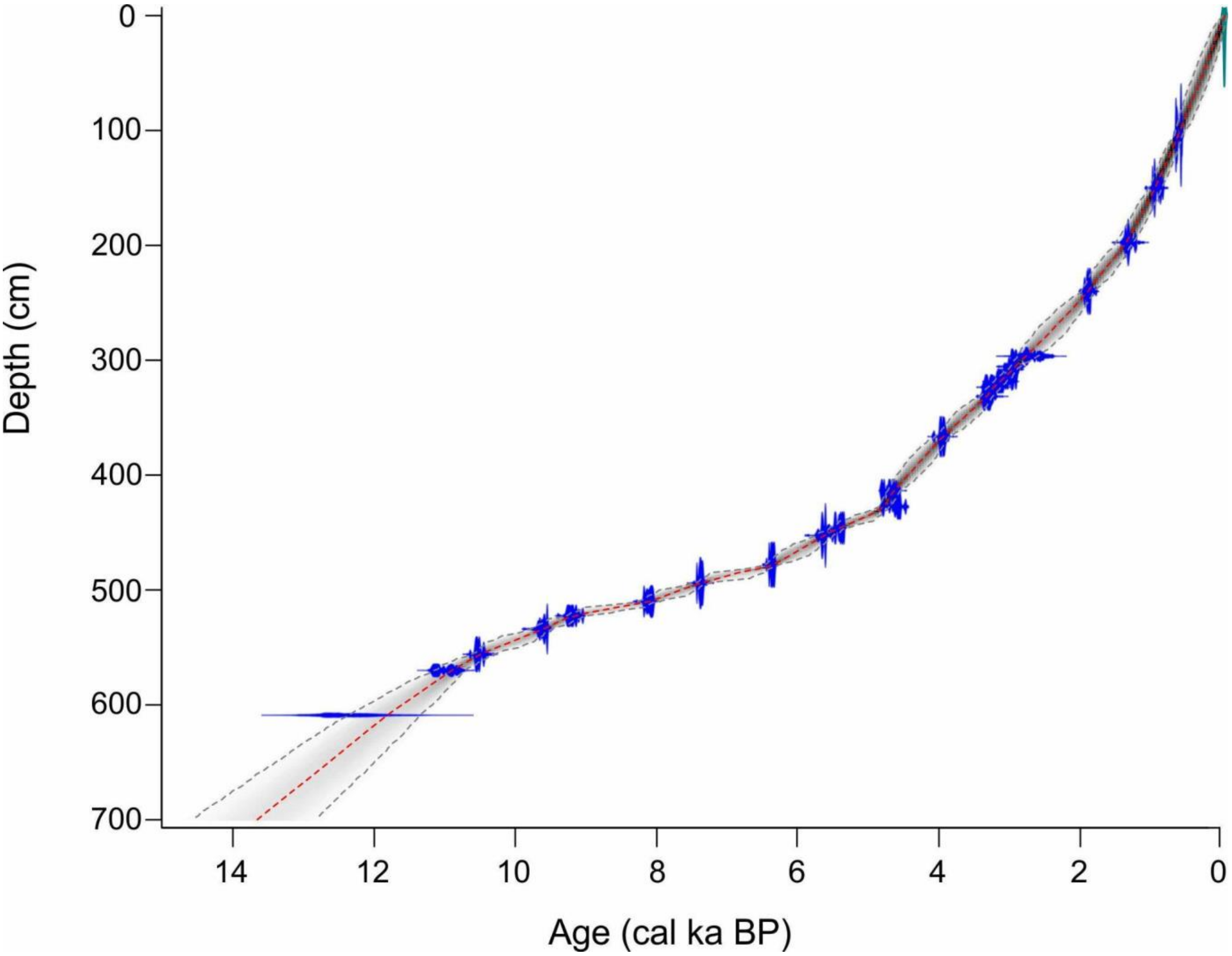

Supplementary Figure 2. Age-depth model of the composite core.

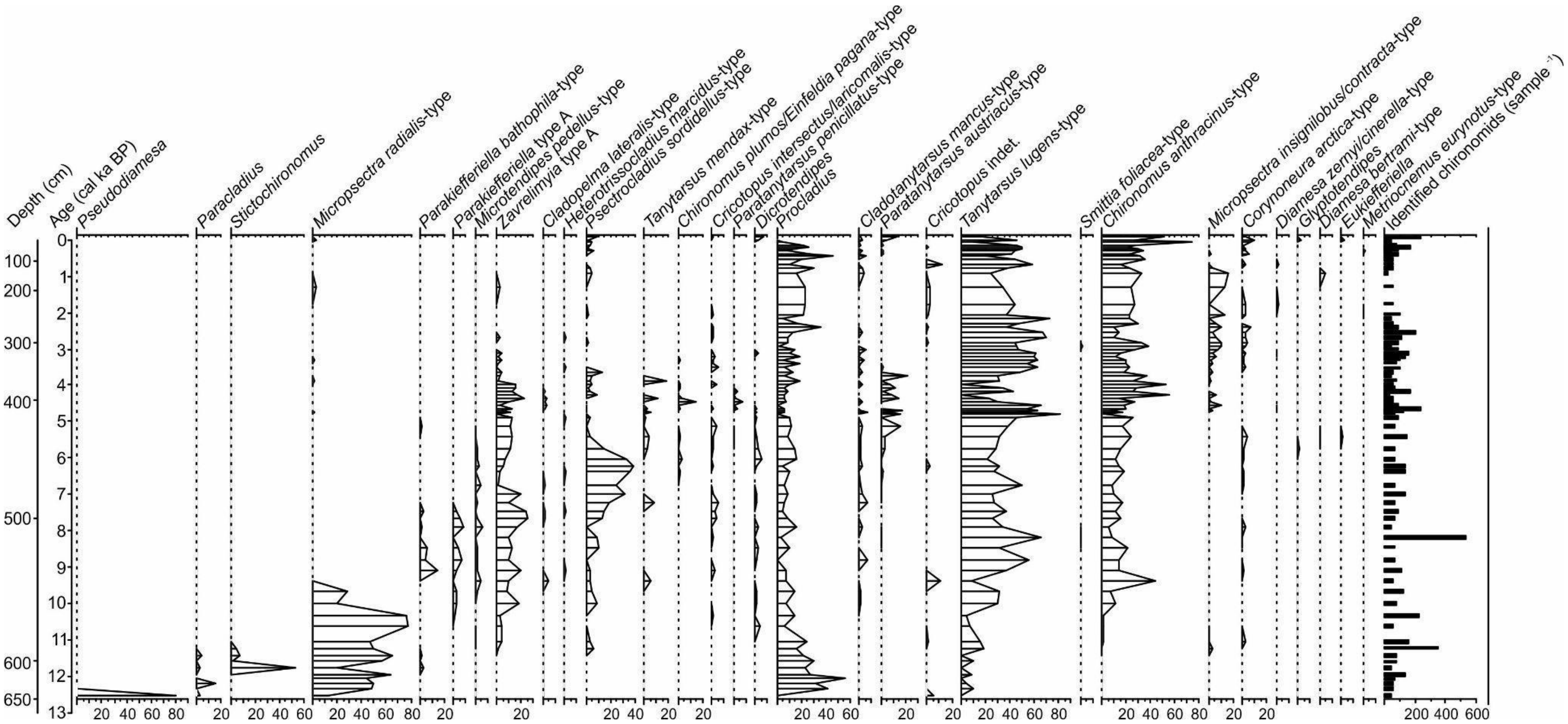

Supplementary Figure 3. Chironomid record from Sulsseewli. Chironomid taxa are plotted as percentages.

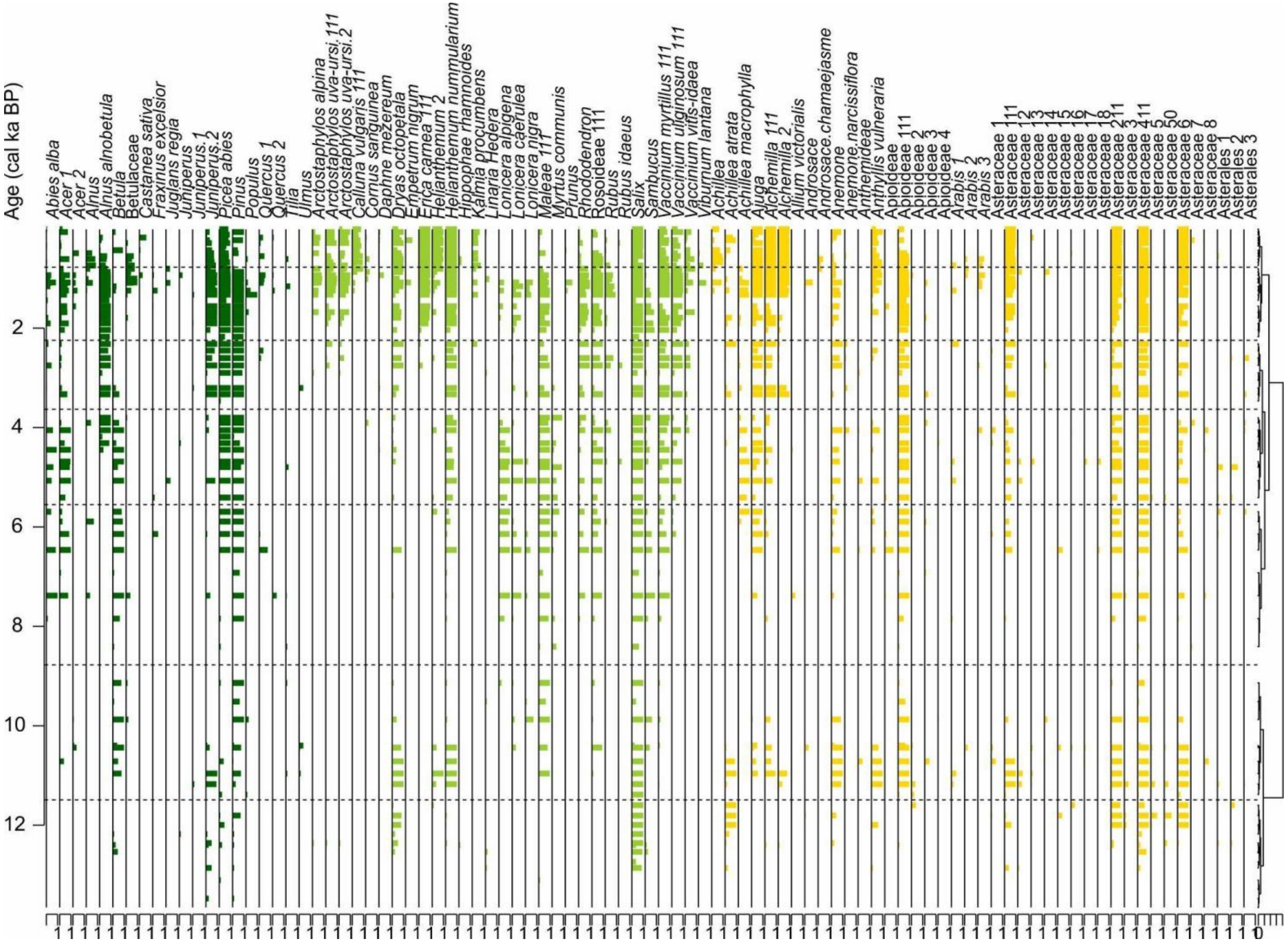

Supplementary Figure 4. Proportion of weighted PCR replicates of plant *sed*aDNA plotted against age with CONISS statistical zonations.

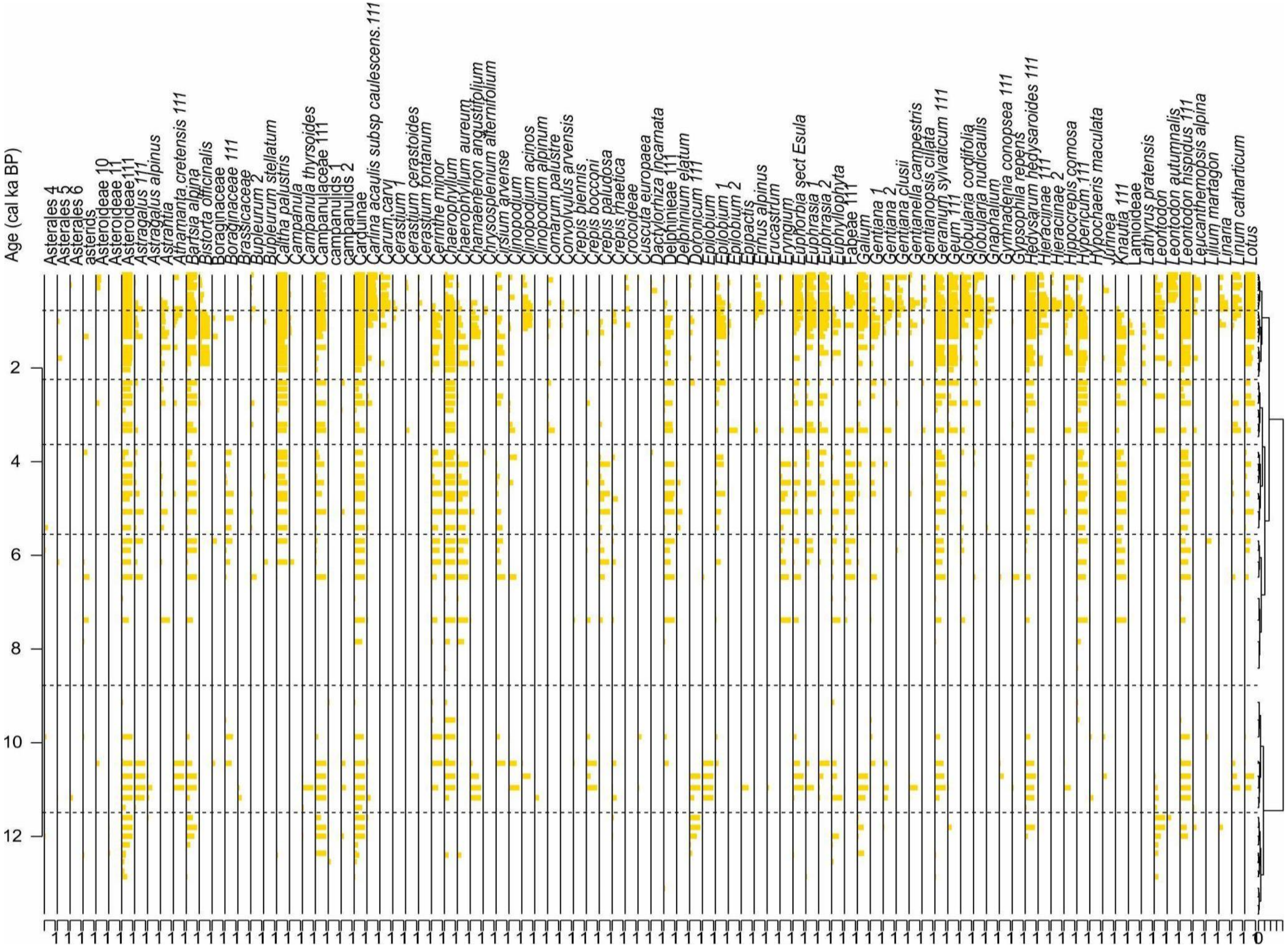
Age (cal ka BP)
2
4
6
8
10
12
Asterales 4
Asterales 5
Asterales 6
asterids
Asteroideae 10
Asteroideae 11
Asteroideae111
Astragalus 111
Astragalus alpinus
Astrantia
Athamanta cretensis 111
Bartsia alpina
Bistorta officinalis
Boraginaceae
Boraginaceae 111
Brassicaceae
Bupleurum 2
Bupleurum stellatum
Caltha palustris
Campanula
Campanula thyrsoides
Campanulaceae 111
campanulids 1
campanulids 2
Carduinae
Carlina acaulis subsp caulescens.111
Carum carvi
Cerastium 1
Cerastium cerastoides
Cerastium fontanum
Cerinthe minor
Chaerophyllum
Chaerophyllum aureum
Chamaenerion angustifolium
Chrysosplenium alternifolium
Cirsium arvense
Clinopodium acinos
Clinopodium alpinum
Comarum palustre
Convolvulus arvensis
Crepis biennis
Crepis bocconi
Crepis paludosa
Crepis rhaetica
Crocoideae
Cuscuta europaea
Dactylorhiza incarnata
Delphinieae 111
Delphinium elatum
Doronicum 111
Epilobium
Epilobium 1
Epilobium 2
Epipactis
Erinus alpinus
Erucastrum
Eryngium
Euphorbia sect Esula
Euphrasia 1
Euphrasia 2
Euphyllophyta
Fabeae 111
Galium
Gentiana 1
Gentiana 2
Gentiana clusii
Gentianella campestris
Gentianopsis ciliata
Geranium sylvaticum 111
Geum 111
Globularia cordifolia
Globularia nudicaulis
Gnaphalium
Gymnadenia conopsea 111
Gypsophila repens
Hedysarum hedysaroides 111
Hieraciinae 111
Hieraciinae 2
Hippocrepis comosa
Hypericum 111
Hypochaeris maculata
Jurinea
Knautia 111
Lamioideae
Lathyrus pratensis
Leontodon
Leontodon autumnalis
Leontodon hispidus 111
Leucanthemopsis alpina
Lilium martagon
Linaria
Linum catharticum
Lotus
0

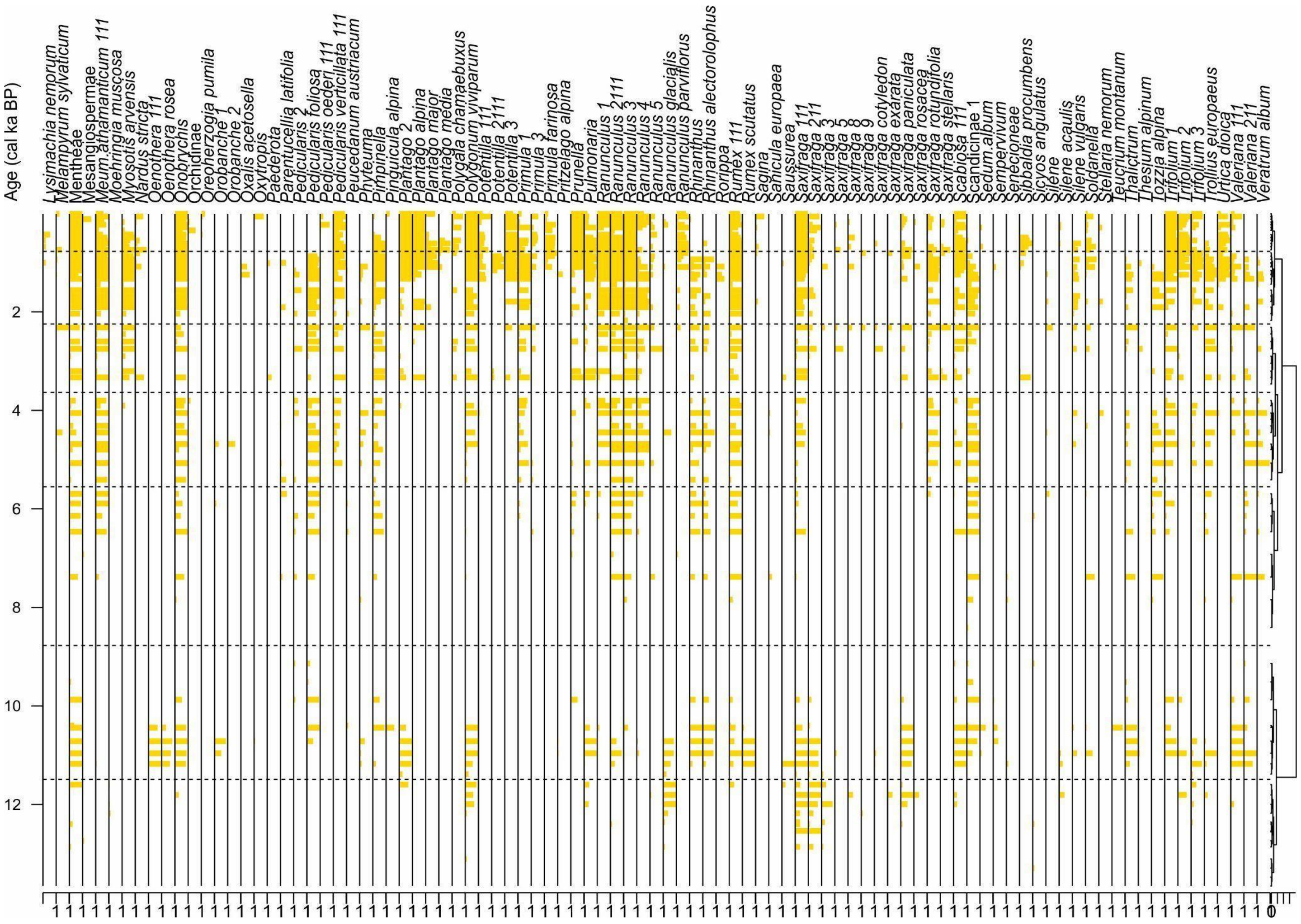
Age (cal ka BP)
2
4
6
8
10
12
Lysimachia nemorum
Melampyrum sylvaticum
Mentheae
Mesangiospermae
Meum athamanticum 111
Moehringia muscosa
Myosotis arvensis
Nardus stricta
Oenothera 111
Oenothera rosea
Onobrychis
Orchidinae
Oreoherzogia pumila
Orobanche 1
Orobanche 2
Oxalis acetosella
Oxytropis
Paederota
Parentucellia latifolia
Pedicularis 2
Pedicularis foliosa
Pedicularis oederi 111
Pedicularis verticillata 111
Peucedanum austriacum
Phyteuma
Pimpinella
Pinguicula alpina
Plantago 2
Plantago alpina
Plantago major
Plantago media
Polygala chamaebuxus
Polygonum viviparum
Potentilla 111
Potentilla 2111
Potentilla 3
Primula 1
Primula 3
Primula farinosa
Pritzelago alpina
Prunella
Pulmonaria
Ranunculus 1
Ranunculus 2111
Ranunculus 3
Ranunculus 4
Ranunculus 5
Ranunculus glacialis
Ranunculus parviflorus
Rhinanthus
Rhinanthus alectorolophus
Rorippa
Rumex 111
Rumex scutatus
Sagina
Sanicula europaea
Saussurea
Saxifraga 111
Saxifraga 211
Saxifraga 3
Saxifraga 5
Saxifraga 8
Saxifraga 9
Saxifraga cotyledon
Saxifraga exarata
Saxifraga paniculata
Saxifraga rosacea
Saxifraga rotundifolia
Saxifraga stellaris
Scabiosa 111
Scandicinae 1
Sedum album
Sempervivum
Senecioneae
Sibbaldia procumbens
Sicyos angulatus
Silene
Silene acaulis
Silene vulgaris
Soldanella
Stellaria nemorum
Teucrium montanum
Thalictrum
Thesium alpinum
Tozzia alpina
Trifolium 1
Trifolium 2
Trifolium 3
Trollius europaeus
Urtica dioica
Valeriana 111
Valeriana 211
Veratrum album

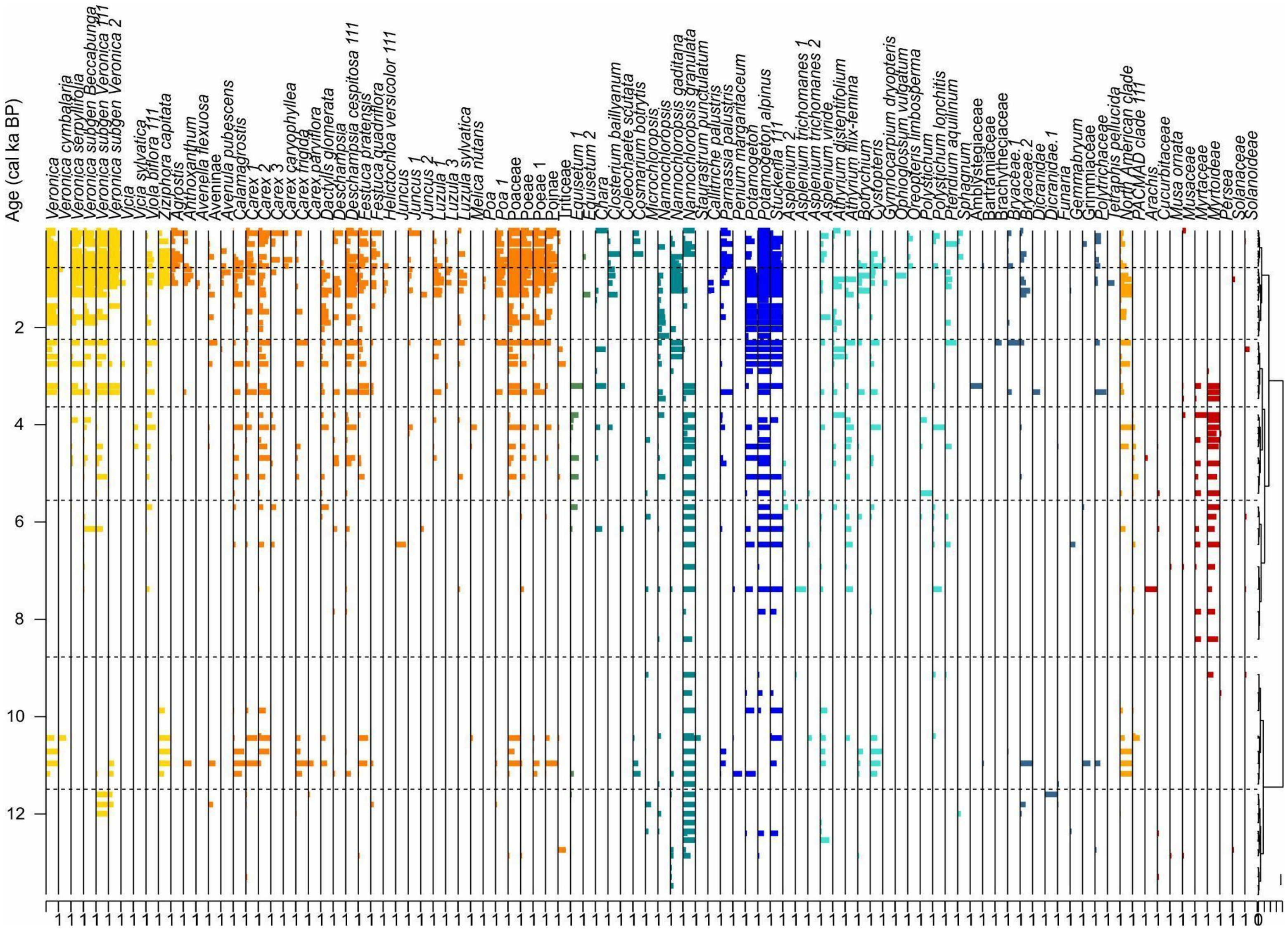
Age (cal ka BP)
Veronica
Veronica cymbalaria
Veronica serpyllifolia
Veronica subgen Beccabunga
Veronica subgen Veronica 111
Veronica subgen Veronica 2
Vicia
Vicia sylvatica
Viola biflora 111
Ziziphora capitata
Agrostis
Anthoxanthum
Avenella flexuosa
Aveninae
Avenula pubescens
Calamagrostis
Carex 1
Carex 2
Carex 3
Carex caryophyllea
Carex frigida
Carex parviflora
Dactylis glomerata
Deschampsia
Deschampsia cespitosa 111
Festuca pratensis
Festuca quadriflora
Helictochloa versicolor 111
Juncus
Juncus 1
Juncus 2
Luzula 1
Luzula 3
Luzula sylvatica
Melica nutans
Poa
Poa 1
Poaceae
Poeae
Poeae 1
Poinae
Triticeae
Equisetum 1
Equisetum 2
Chara
Closterium baillyanum
Coleochaete scutata
Cosmarium botrytis
Microchloropsis
Nannochloropsis
Nannochloropsis gaditana
Nannochloropsis granulata
Staurastrum punctulatum
Callitriche palustris
Parnassia palustris
Penium margaritaceum
Potamogeton
Potamogeton alpinus
Stuckenia 111
Asplenium 2
Asplenium trichomanes 1
Asplenium trichomanes 2
Asplenium viride
Athyrium distentifolium
Athyrium filix-femina
Botrychium
Cystopteris
Gymnocarpium dryopteris
Ophioglossum vulgatum
Oreopteris limbosperma
Polystichum
Polystichum lonchitis
Pteridium aquilinum
Sphagnum
Amblystegiaceae
Bartramiaceae
Brachytheciaceae
Bryaceae.1
Bryaceae.2
Dicranidae
Dicranidae.1
Funaria
Gemmabryum
Grimmiaceae
Polytrichaceae
Tetraphis pellucida
North American clade
PACMAD clade 111
Arachis
Cucurbitaceae
Musa ornata
Musaceae
Myrtaceae
Myrtoideae
Persea
Solanaceae
Solanoideae
2
4
6
8
10
12
0

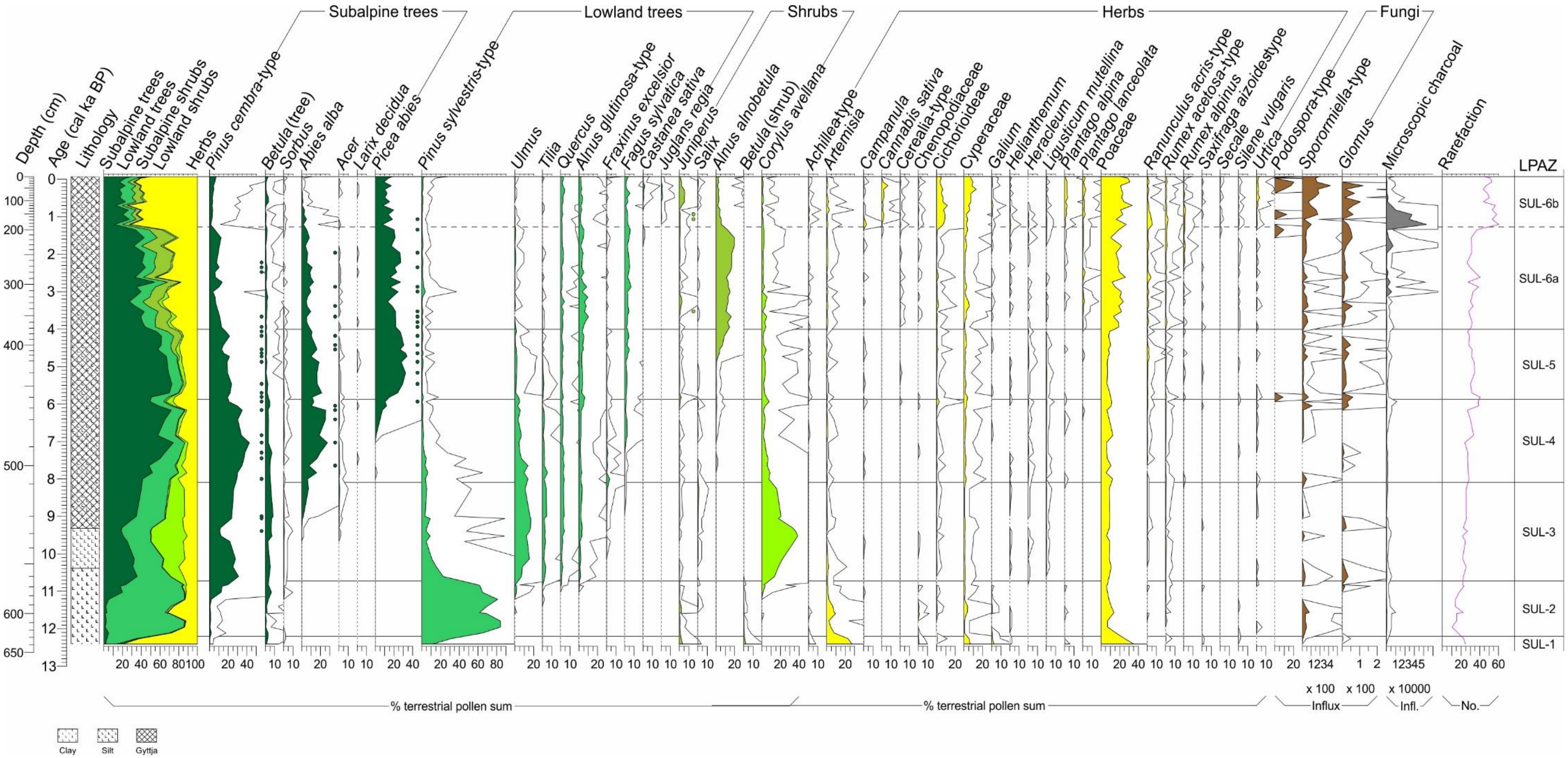

Supplementary Figure 5. Pollen diagram of Sulsseewli based on cores SUL-A and SULB. Age scale and influx are based on the 14C ages from these two cores. Pollen is presented as percentages of terrestrial pollen sum. Microcharcoal and fungi spores are presented as influx values (cm^{-2} $year^{-1}$). LPAZ are the statistically significant zones determined with partitioning using optimal sum of squares and the broken stick method. Rarefaction based pollen diversity is standardized to a count sum of 382.

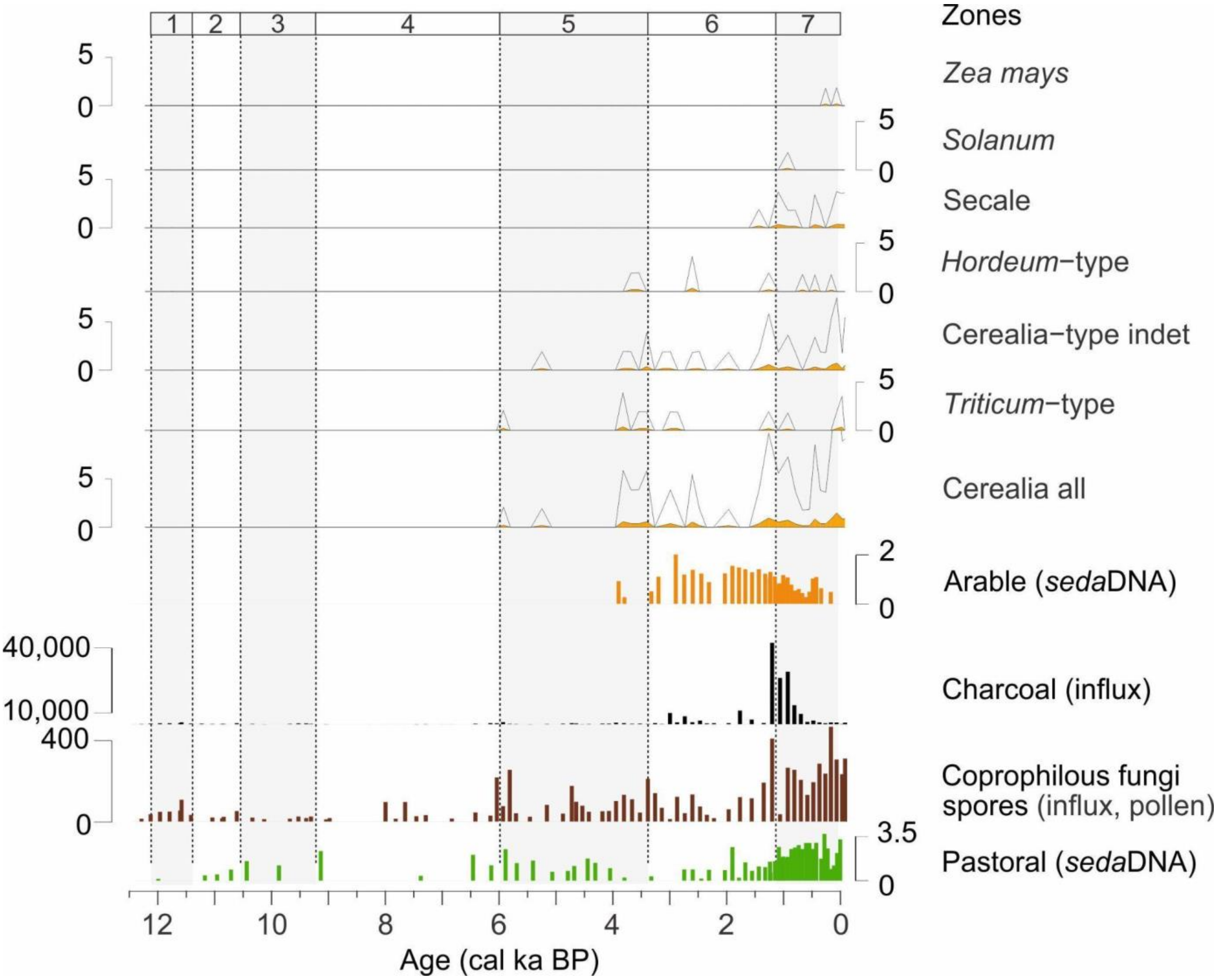

Supplementary Figure 6. Summary of the indicators for human activities in the Sulsseewli sediments. Cerealia pollen sum and pollen types (with 10x exaggeration line), arable *seda*DNA indicators, charcoal, coprophilous fungi spores sum and pastoral *seda*DNA indicators. Zones based on CONISS analysis of the *seda*DNA data are marked in grey and white. Pollen are represented as percentage of terrestrial pollen, spores and charcoal as influx (particles cm-2 year-1) and *seda*DNA as relative abundance index (RAI)

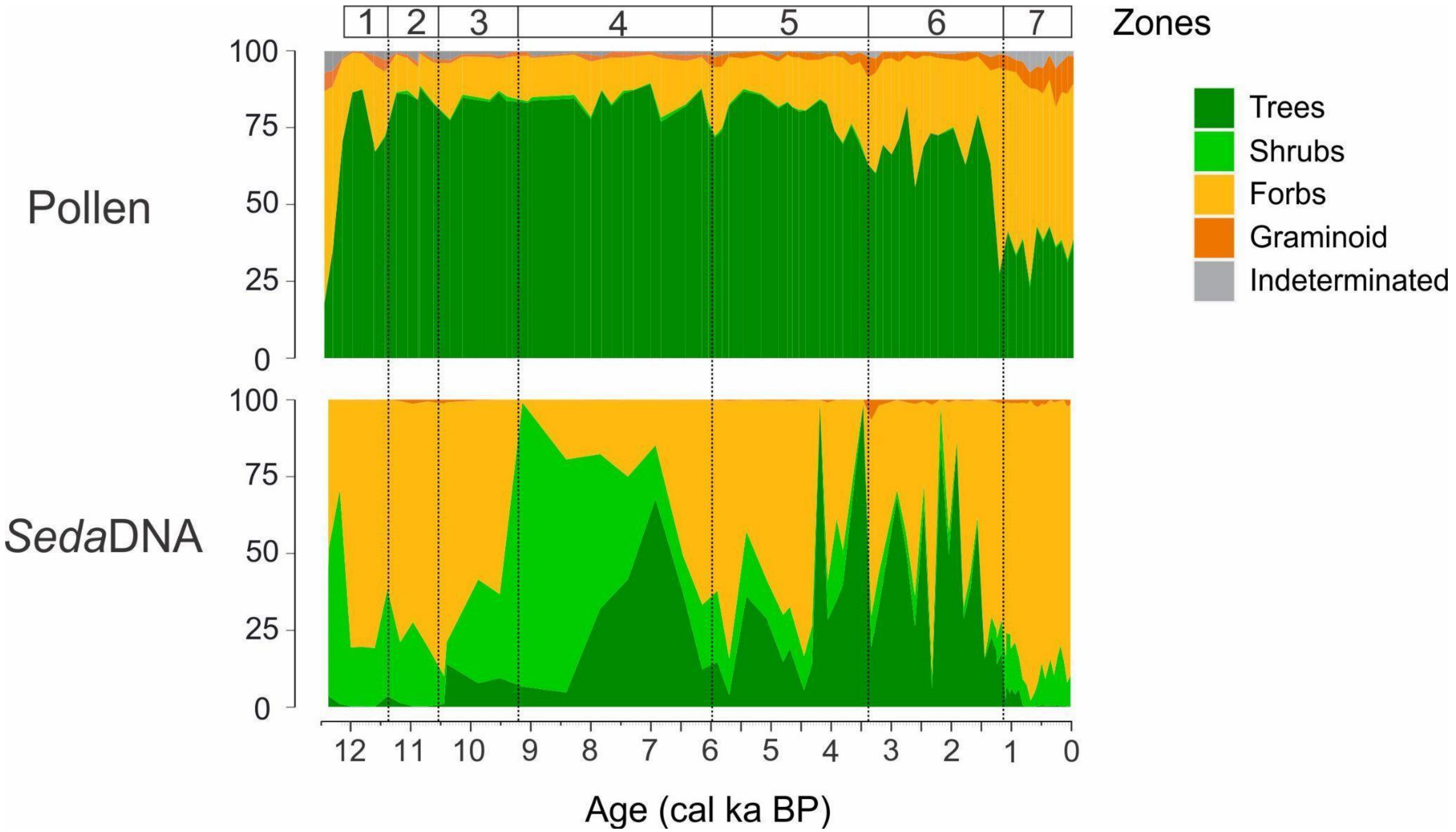

Supplementary Figure 7. Percentages of functional groups inferred by *sed*aDNA (relative abundance index, RAI) and pollen (% of terrestrial pollen). Zones are based on CONISS analysis of the *sed*aDNA data (see Methods section for details)

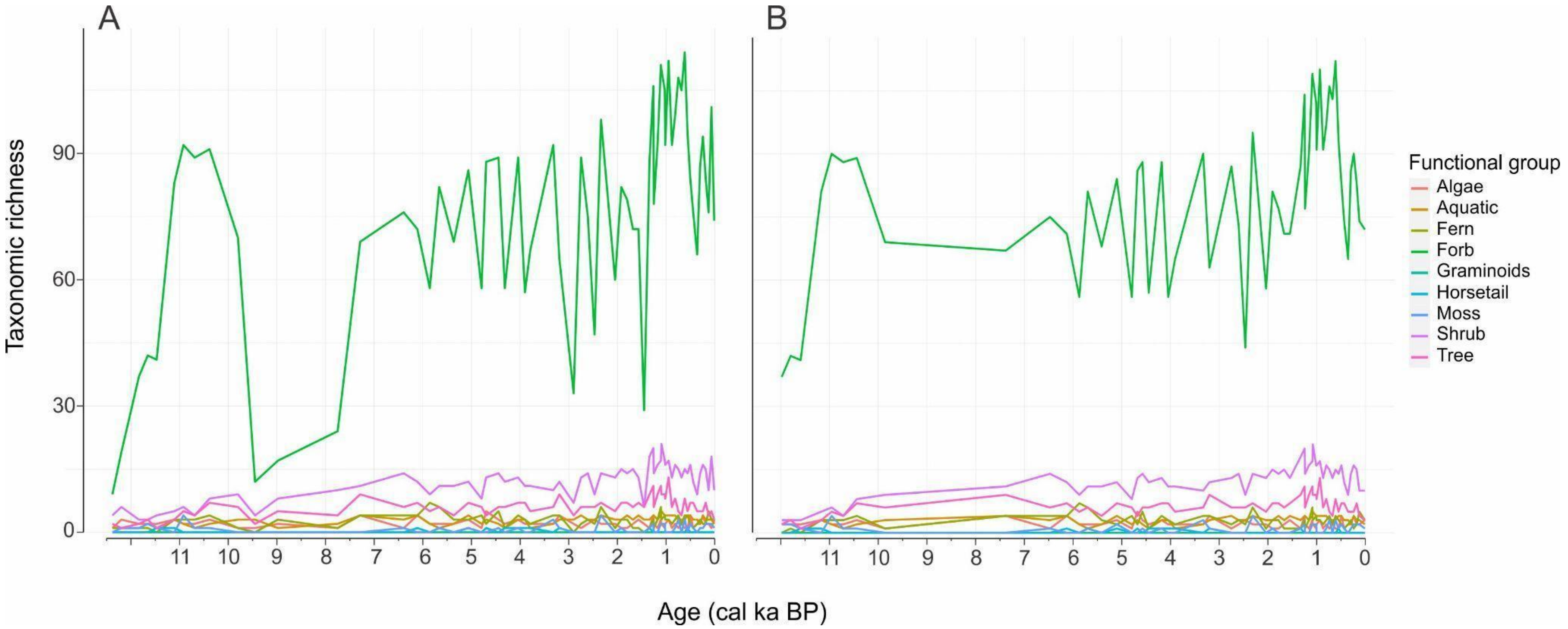

Supplementary Figure 8. Richness of *sed*aDNA within different plant functional groups with the relaxed (A) or strict (B) quality filter.

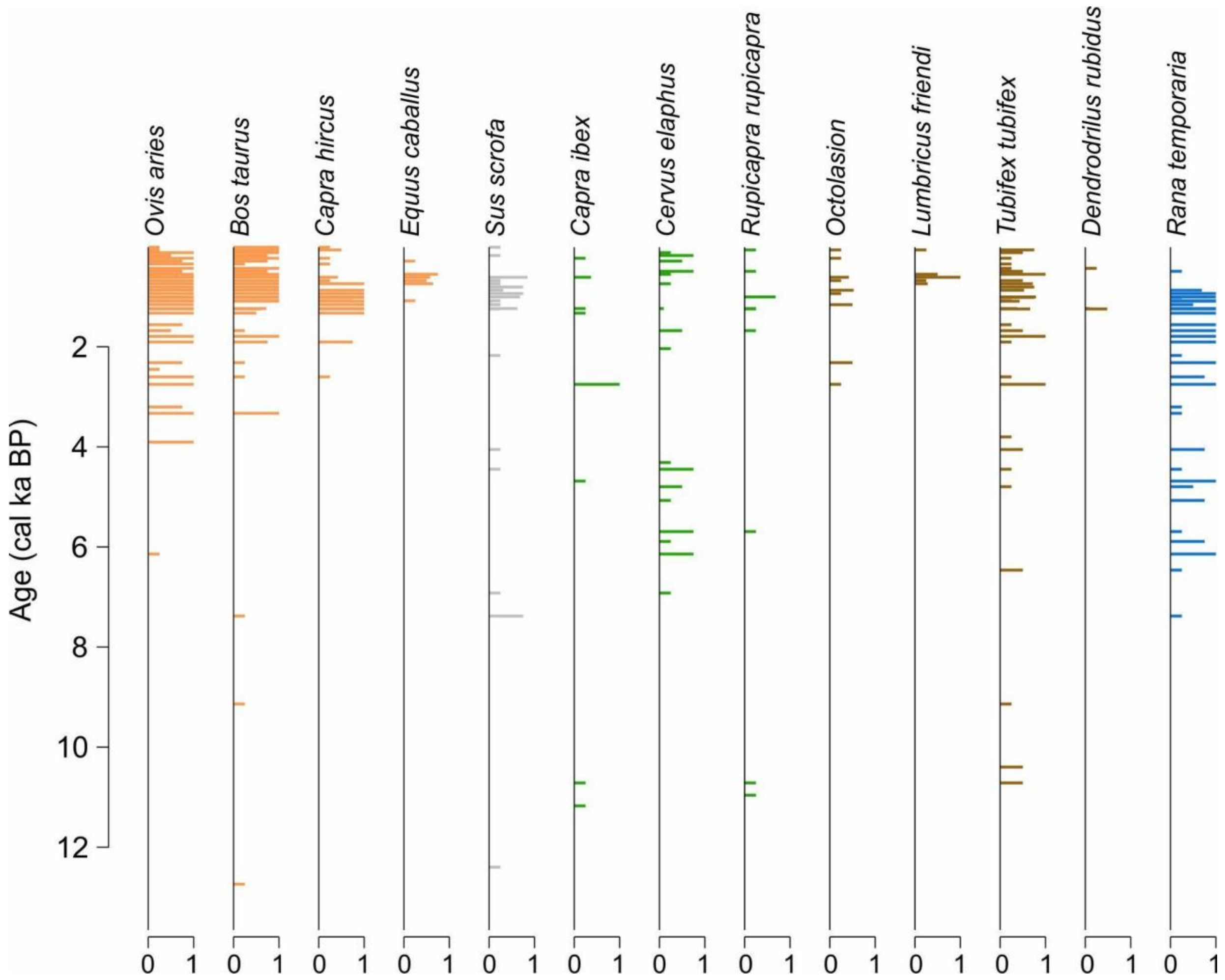

Supplementary Figure 9. All retained animal *sed*aDNA detections. Domesticated taxa are orange (pig (Sus scrofa), which was excluded from analysis, is in grey), wild mammals are in green, non mammal *sed*aDNA records are in brown (invertebrates) and blue (amphibians). *Sed*aDNA is plotted as proportion of weighted PCR replicates.

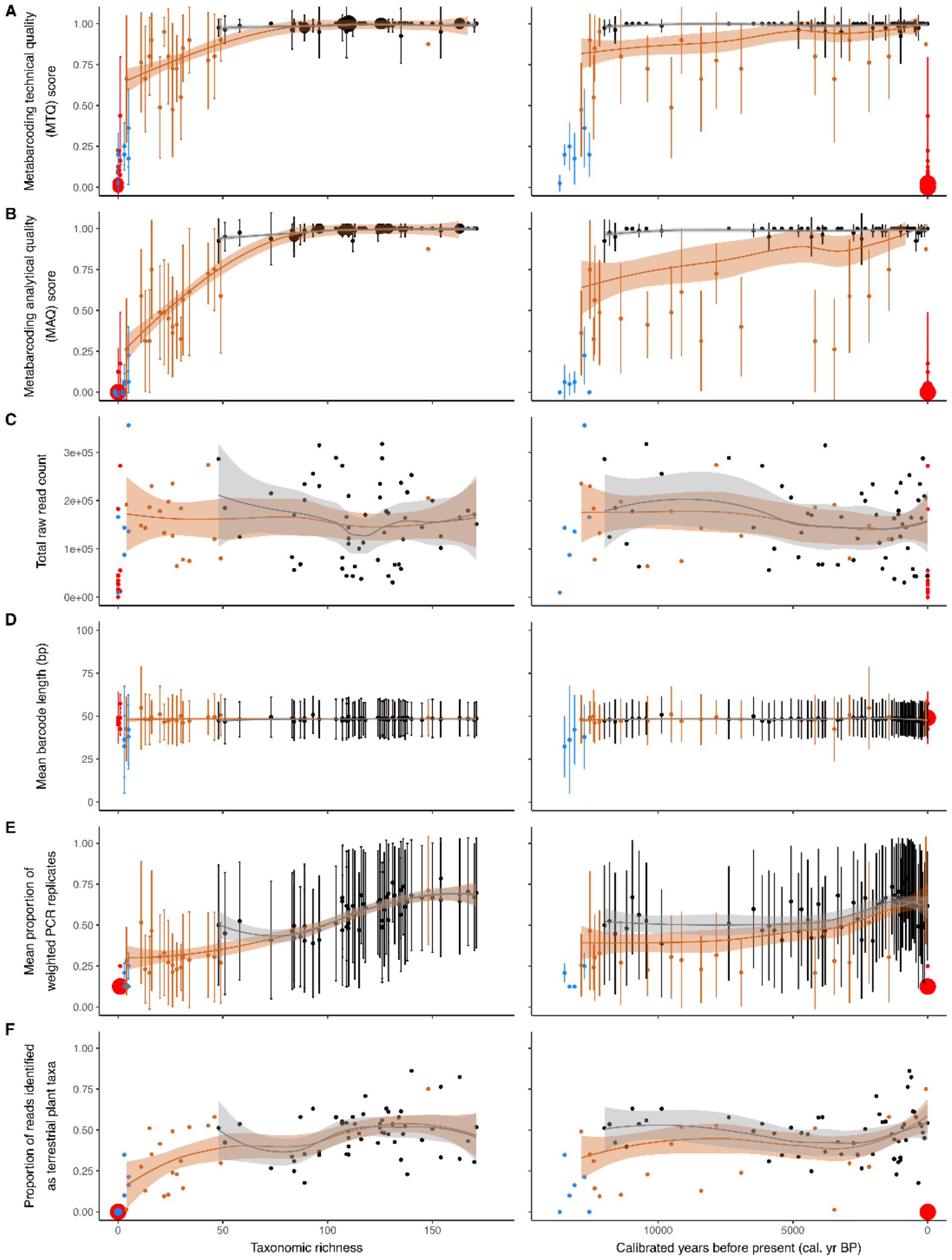

Supplementary Figure 10. Correlations between total taxonomic richness (left) and time (right) against six measures of plant *sed*aDNA data quality: (A) metabarcoding technical quality (MTQ) score, (B) metabarcoding analytical quality (MAQ) score, (C) total raw read count, (D) mean barcode length (base pairs), (E) mean proportion of weighted PCR replicates, (F) proportion of reads identified as terrestrial plant taxa. Data in: black, samples that passed strict quality control; orange, samples that passed relaxed quality control only; blue, samples that failed quality control; red, negative controls. In (A), (B), (D), and (E), points are mean values and bars one standard deviation derived from technical PCR replicates (n=8). Fitted Loess-smoothed lines with 95% confidence intervals are shown for the samples that passed quality control (strict, black; relaxed, orange).

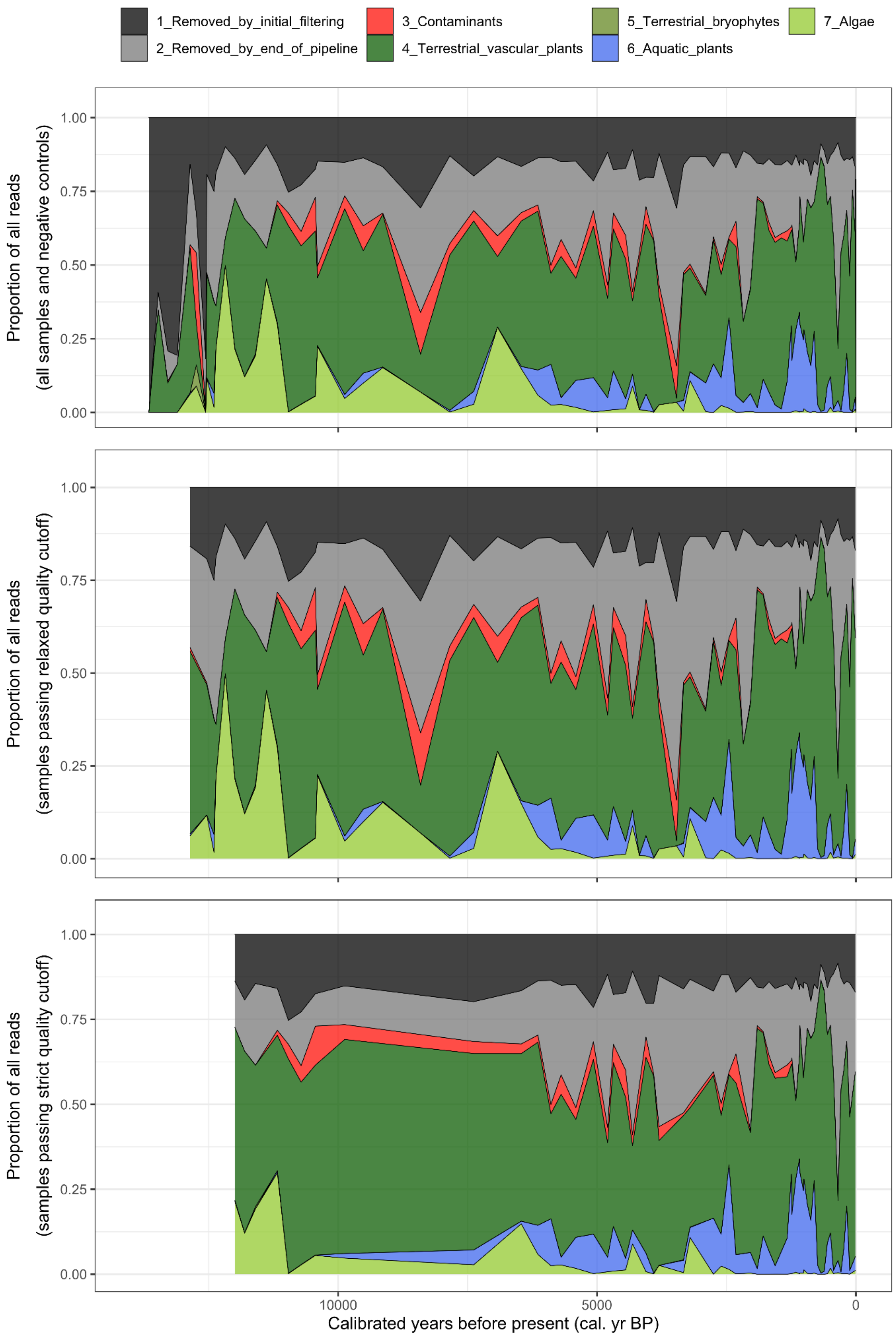

Supplementary Figure 11. Proportion of all reads prior to (upper panel) and after the plant metabarcoding data quality control with either relaxed (middle panel) or strict (lower panel) filtering.

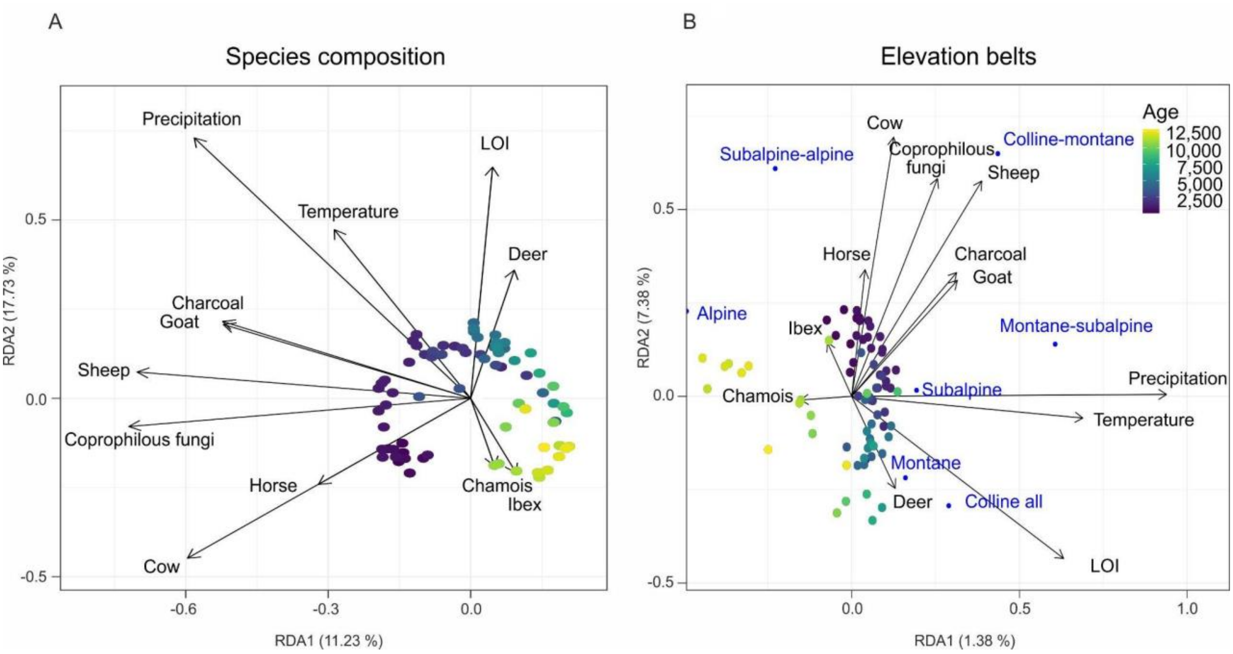

Supplementary Figure 12. Redundancy analysis (RDA) of the plant species composition (A) and altitudinal vegetation belts (B) from *sed*aDNA across the record constrained by 12 explanatory variables representing human activities (coprophilous fungi spores, mammal DNA and microcharcoal) and climatic changes (chironomid-inferred temperatures, CHELSA-TraCE21k v1.0 model-inferred precipitation and organic matter content (LOI)).

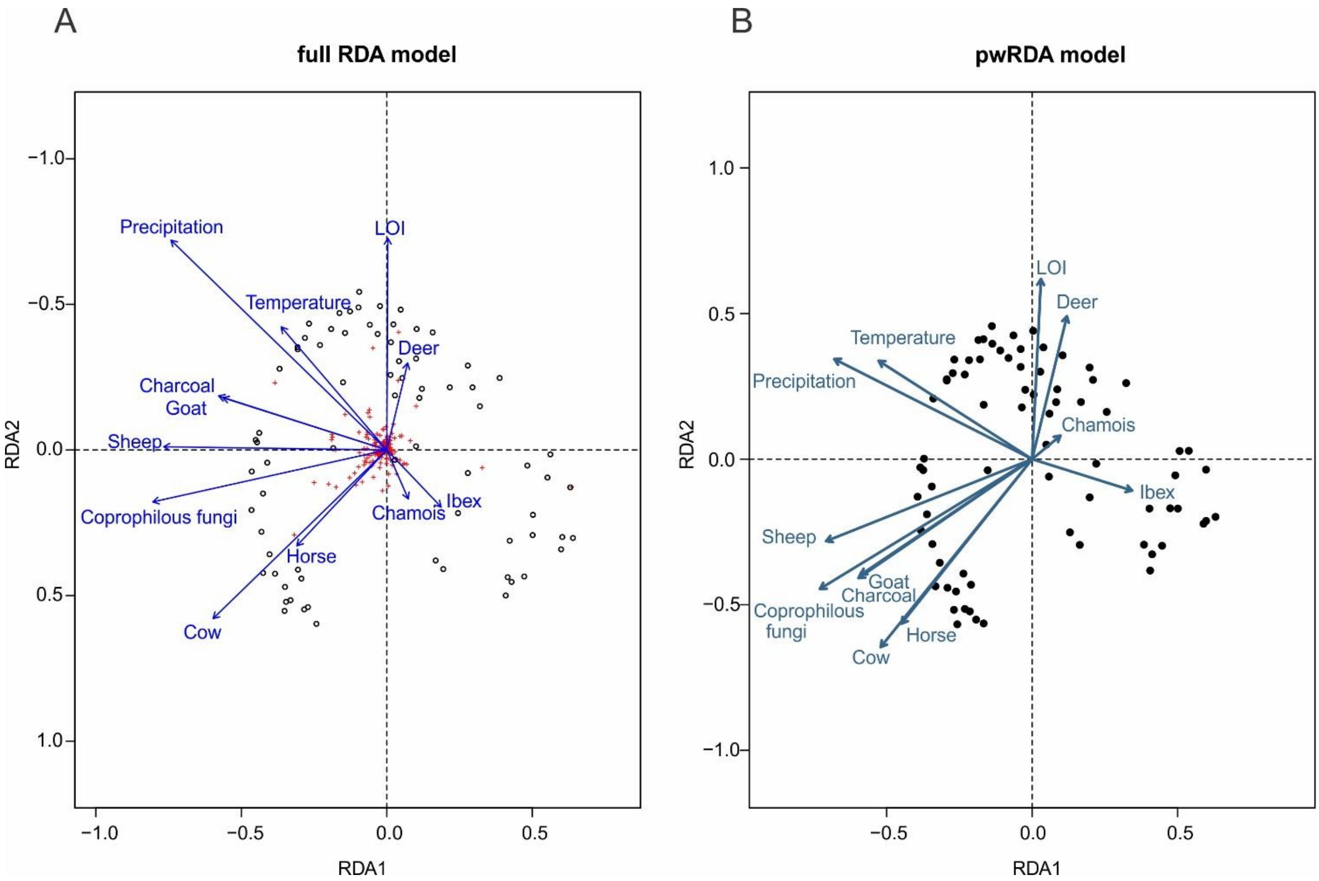

Supplementary Figure 13. To crosscheck the robustness of the RDA analyses of the effect of the 12 explanatory variables on the species composition (Supplementary Figure 12A), we also performed the Piecewise redundancy analysis. Results of this analysis do not differ from those of the original RDA analysis. Full RDA (A) and piecewise RDA (B). Layout is different from Figure 12A due to R package used.

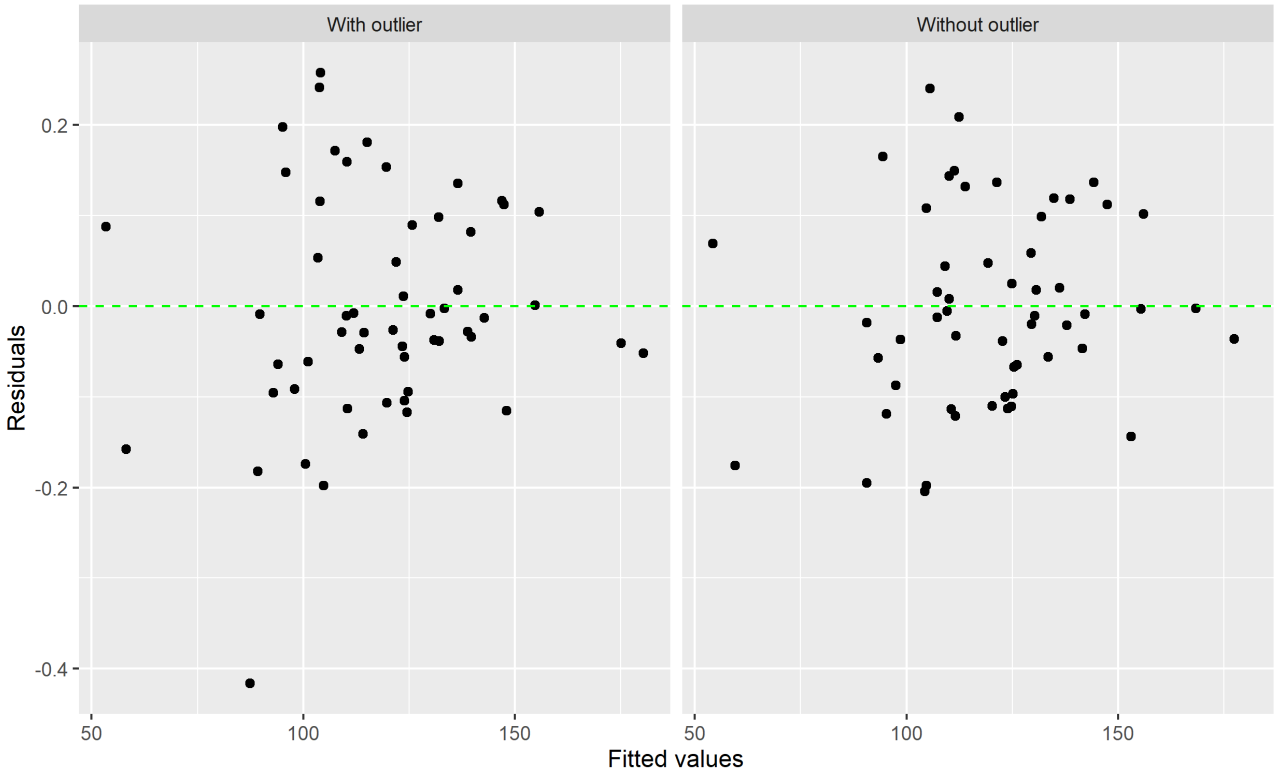

Supplementary Figure 14. Distribution of residuals before and after the removal of the outlier L652 from generalised additive models (GAMs)

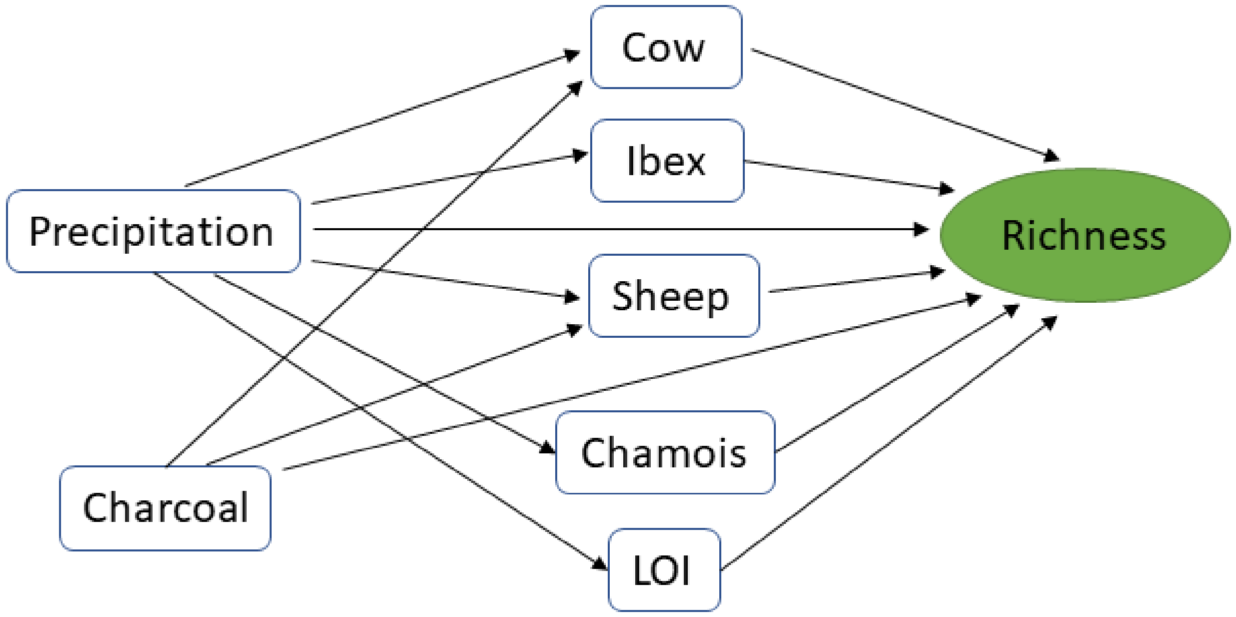

Supplementary Figure 15. Full model used for structural equation model analysis.
