# Supplementary material for: High resolution ancient sedimentary DNA shows that alpine plant diversity is associated with human land use and climate change
Source: Nat Commun. 2022 Nov 4;13:6559. doi: 10.1038/s41467-022-34010-4 (PMC9636257; doi:10.1038/s41467-022-34010-4)
Supplement: Supplementary file 3 — Description of Additional Supplementary Files [file 41467_2022_34010_MOESM3_ESM.pdf]

Description of Additional Supplementary Files

File Name: Supplementary Data 1
Description: TrnL p6loop plant sequences

File Name: Supplementary Data 2
Description: Pollen vs DNA

File Name: Supplementary Data 3
Description: Occurrences and taxonomic assignments of the 16S sequences

File Name: Supplementary Data 4
Description: PhyloAlps database fasta

File Name: Supplementary Data 5
Description: Age and depth of the sedaDNA samples

File Name: Supplementary Data 6
Description: Age and depth of the pollen samples

File Name: Supplementary Data 7
Description: Primer tag to PCR replicate lookup

File Name: Supplementary Data 8
Description: Proportions of weighted PCR replicates of the taxa found in the controls of the plant sedaDNA analyses

File Name: Supplementary Data 9
Description: Proportion of weighted PCR replicates of the sedaDNA mammal taxa found in the controls

File Name: Supplementary Data 10
Description: Statistical results of the RDA analyses

File Name: Supplementary Data 11
Description: Chironomid assemblages from the unteres Sulssweewli record as shown in Supplementary information.

File Name: Supplementary Data 12
Description: Chironomid-inferred mean July air temperatures (°C) for the unteres Sulsseewli record.
