# Supplementary material for: High resolution ancient sedimentary DNA shows that alpine plant diversity is associated with human land use and climate change
Source: Nat Commun. 2022 Nov 4;13:6559. doi: 10.1038/s41467-022-34010-4 (PMC9636257; doi:10.1038/s41467-022-34010-4)
Supplement: Supplementary file 5 — Reporting Summary [file 41467_2022_34010_MOESM5_ESM.pdf]

nature portfolio

Corresponding author(s): Sandra Garcés-Pastor

Last updated by author(s): Sep 8, 2022

Reporting Summary

Nature Portfolio wishes to improve the reproducibility of the work that we publish. This form provides structure for consistency and transparency in reporting. For further information on Nature Portfolio policies, see our Editorial Policies and the Editorial Policy Checklist.

Statistics

For all statistical analyses, confirm that the following items are present in the figure legend, table legend, main text, or Methods section.

n/a	Confirmed	
☐	☒	The exact sample size (*n*) for each experimental group/condition, given as a discrete number and unit of measurement
☐	☒	A statement on whether measurements were taken from distinct samples or whether the same sample was measured repeatedly
☐	☒	The statistical test(s) used AND whether they are one- or two-sided<br>*Only common tests should be described solely by name; describe more complex techniques in the Methods section.*
☒	☐	A description of all covariates tested
☒	☐	A description of any assumptions or corrections, such as tests of normality and adjustment for multiple comparisons
☐	☒	A full description of the statistical parameters including central tendency (e.g. means) or other basic estimates (e.g. regression coefficient) AND variation (e.g. standard deviation) or associated estimates of uncertainty (e.g. confidence intervals)
☐	☒	For null hypothesis testing, the test statistic (e.g. *F*, *t*, *r*) with confidence intervals, effect sizes, degrees of freedom and *P* value noted<br>*Give P values as exact values whenever suitable.*
☒	☐	For Bayesian analysis, information on the choice of priors and Markov chain Monte Carlo settings
☒	☐	For hierarchical and complex designs, identification of the appropriate level for tests and full reporting of outcomes
☒	☐	Estimates of effect sizes (e.g. Cohen's *d*, Pearson's *r*), indicating how they were calculated

Our web collection on statistics for biologists contains articles on many of the points above.

Software and code

Policy information about availability of computer code

Data collection	No software was used for data collection
Data analysis	OBITools software package was used to analyse the sequence data.<br>Plots were made with R v3.4.2 using the Vegan v.2.5-7, Rioja v.0.9-26 and Ggplot2 v.3.3.5 packages.<br>RDA and CONISS were also calculated in R, using the Vegan v.2.5-7, Rioja v.0.9-26, segRDA v.1.0.2 and piecewiseSEM v.2.1.2 packages.

For manuscripts utilizing custom algorithms or software that are central to the research but not yet described in published literature, software must be made available to editors and reviewers. We strongly encourage code deposition in a community repository (e.g. GitHub). See the Nature Portfolio guidelines for submitting code & software for further information.

Data

Policy information about availability of data

All manuscripts must include a data availability statement. This statement should provide the following information, where applicable:

- Accession codes, unique identifiers, or web links for publicly available datasets
- A description of any restrictions on data availability
- For clinical datasets or third party data, please ensure that the statement adheres to our policy

The raw DNA sequence data generated in this study have been deposited in the European Nucleotide Archive (ENA) under BioProject accession code PRJEB52290. The unfiltered OBItools output tsv files used in this study are available in the Dryad (datadryad.org, doi: https://doi.org/10.5061/dryad.7wm37pvx5), pollen and charcoal data are available in the Neotoma database under the accession codes https://doi.org/10.21233/NM0Y-GM04 and https://doi.org/10.21233/J8P9-G487 (https://www.neotomadb.org).

Field-specific reporting

Please select the one below that is the best fit for your research. If you are not sure, read the appropriate sections before making your selection.

☐ Life sciences ☐ Behavioural & social sciences ☒ Ecological, evolutionary & environmental sciences

For a reference copy of the document with all sections, see nature.com/documents/nr-reporting-summary-flat.pdf

Ecological, evolutionary & environmental sciences study design

All studies must disclose on these points even when the disclosure is negative.

Study description	Here, we reconstruct the response of the vegetation around Lake Sulsseewli to climate and human activities over the past 12,000 years. We used a multiproxy approach consisting of plant sedaDNA, pollen, fossil chironomids for summer temperature reconstruction, precipitation, geochemical proxies, and multiple independent indicators of human activity, that included microscopic charcoal (reflecting fire activity) and grazing indicators (coprophilous fungi spores and mammalian sedaDNA).
Research sample	Four parallel cores (SUL A, B, C, D) were retrieved from Sulsseewli. Cores A, B and C were opened by longitudinal splitting. One half was used for proxy subsampling, and the other half was used for photography. Lithostratigraphic markers were used to correlate cores A, B and C resulting in a continuous composite sequence of 716 cm
Sampling strategy	The parallel cores were sampled for sedimentary DNA, pollen, chironomids, loss-of-ignition and macrofossils. Twenty-three plant macrofossil remains were sampled in cores SUL A, B, C, and radiocarbon-dated using accelerator mass spectrometry (AMS).<br>A total of 75 samples were sampled from a correlated composite section of cores SUL A and B for chironomid, pollen, spores and microscopic charcoal analyses. Finally, 80 samples were obtained from core SUL C in the ancient DNA lab at The Arctic University Museum of Norway in Tromsø. A 10 cm resolution was determined in order to have an homogeneous record of the sedimentary core.
Data collection	Inger Greve Alsos, Christoph Schwörer, and Fabian Rey did the fieldwork; Sandra Garcés-Pastor did the ancient DNA laboratory work with input from Inger Greve Alsos and Peter D. Heintzman; Youri Lammers performed the bioinformatic pipeline. Plant sequences were curated by Sandra Garcés-Pastor and Peter D. Heintzman curated the mammals. Tomasz Goslar performed radiocarbon dating; Christoph Schwörer built composite cores and Sandra Garcés-Pastor performed age-depth modeling with input from Christoph Schwörer and Oliver Heiri; Fabian Rey performed pollen, charcoal and non-pollen palynomorphs analysis. Martina Heer and Astrid Rutzer performed chironomid analysis supervised by Oliver Heiri, who also built the temperature reconstruction. Dirk N. Karger and Loïc Pellissier performed the precipitation time series for the lake location. All data was stored in csv and excel files.
Timing and spatial scale	Coring opening, sampling and extractions occurred on February-March 2019. All DNA samples were sampled and extracted at the same time, the same applies for pollen and chironomids.
Data exclusions	During the analysis 8 samples for plants with low quality were removed, which had technical quality (MTQ) scores <0.45 and/or analytical quality (MAQ) scores <0.175. All samples, including the ones that were removed, are included in the raw data.
Reproducibility	8 PCR amplification replicates were performed and any DNA samples with poor replication were discarded.
Randomization	Randomization is not applicable to this study. Our study is based on temporal data.
Blinding	We did not use different treatment for our samples, therefore blinding was not used.

Did the study involve field work? ☒ Yes ☐ No

Field work, collection and transport

Field conditions	Sulsseewli is located in the subalpine vegetation zone below the present treeline (1,921 m a.s.l.), this allows us to study past vegetation trends. This region has a cool temperate climate with mild summers (10 ºC July) and cool winters (-5 ºC January).
Location	Sulsseewli is a small lake (2 ha) in the Bernese Alps (northern Swiss Alps), located in the subalpine vegetation zone below the present treeline (Figure 1, 46.617639° N, 7.864028° E; 1,921 m a.s.l.).
Access & import/export	The lake was accessed by car. Four parallel sedimentary cores were extracted and transported to the Institute of Plant Sciences & Oeschger Centre for Climate Change Research (University of Bern, Switzerland). Cores A and B were stored at the University of Bern, while cores C and D were transported with a refrigerated truck to Norway and stored at the The Arctic University Museum of Norway in Tromsø (TMU, Norway).
Disturbance	No disturbance occurred.

Reporting for specific materials, systems and methods

We require information from authors about some types of materials, experimental systems and methods used in many studies. Here, indicate whether each material, system or method listed is relevant to your study. If you are not sure if a list item applies to your research, read the appropriate section before selecting a response.

Materials & experimental systems

n/a	Involved in the study
☒	☐ Antibodies
☒	☐ Eukaryotic cell lines
☒	☐ Palaeontology and archaeology
☒	☐ Animals and other organisms
☒	☐ Human research participants
☒	☐ Clinical data
☒	☐ Dual use research of concern

Methods

n/a	Involved in the study
☒	☐ ChIP-seq
☒	☐ Flow cytometry
☒	☐ MRI-based neuroimaging
